# The Clinical Value of PET with Amino Acid Tracers for Gliomas WHO Grade II

**DOI:** 10.1155/2011/372509

**Published:** 2011-04-28

**Authors:** Anja Smits, Brigitta G. Baumert

**Affiliations:** ^1^Department of Neuroscience, Neurology, University Hospital, Uppsala University, 751 85 Uppsala, Sweden; ^2^Department of Radiation-Oncology (MAASTRO), GROW (School for Oncology and Developmental Biology), Maastricht University Medical Centre (MUMC), 6202 Maastricht, The Netherlands

## Abstract

The clinical management of adults with low-grade gliomas (LGGs) remains a challenge. There is no curative treatment, and management of individual patients is a matter of deciding optimal timing as well as right treatment modality. In addition to conventional imaging techniques, positron emission tomography (PET) with amino acid tracers can facilitate diagnostic and therapeutic procedures. In this paper, the clinical applications of PET with amino acid tracers ^11^C-methyl-L-methionine (MET) and ^18^F-fluoro-ethyl-L-tyrosine (FET) for patients with LGG are summarized. We also discuss the value of PET for the long-term followup of this patient group. Monitoring metabolic activity by PET in individual patients during course of disease will provide insight in the biological behavior and evolution of these tumors. As such, spatial changes in tumor activity over time, including shifts of hot-spot regions within the tumor, may reflect intratumoral heterogeneity and correlate to clinical parameters.

## 1. Low-Grade Gliomas

### 1.1. Introduction

Low-grade gliomas (LGGs) in adults are brain tumors affecting otherwise healthy people with an average age of around 40 years at the time of diagnosis. The common histological types of LGGs are astrocytomas, oligodendrogliomas, and oligoastrocytomas of malignancy grade II according to the WHO classification [1]. These are poorly circumscribed diffusely infiltrative tumors with a preferential localization in or close to eloquent areas [2]. 

The most frequent presenting symptoms are seizures. Around 65–90% of all patients present with seizures, and epilepsy may be the only symptom for months or years in the initial phase of disease [3]. The median survival of patients with LGGs is 5–10 years, but clinical outcome varies considerably [4]. For some patients, the disease has an indolent course, whereas others experience rapid progression from disease presentation [5]. Patients with astrocytomas carry the highest risk for malignant transformation, their overall 5-year survival has been estimated around 50% [6]. Patients with oligodendrogliomas have a more favorable prognosis with a 5-year survival of 70–80% [4, 7]. It is generally believed that in most patients, if not all, the tumor will eventually transform into a malignant glioma with fatal outcome. 

### 1.2. Clinical Management

Treatment is unsatisfactory and there are still controversies in the clinical management of patients with LGGs [8]. No randomized studies are available that have proven the role of surgery, but the consensus today is that radical tumor resection is associated with a favorable outcome [7, 9, 10]. Two large prospective trials on radiotherapy in LGGs have failed to show a radiotherapeutic dose response [11–13], with reduced quality of life after high-dose radiotherapy compared to low dose [14]. A randomized trial on the optimal timing of radiotherapy showed a longer symptom-free survival by immediate postoperative radiotherapy but no overall survival benefit compared to radiotherapy at the time of progression [15]. Chemotherapy as initial treatment after surgery may be effective and considered for high-risk patients, including patients with large tumor volumes and incomplete resections [16–19]. In general, the chemosensitivity of oligodendroglial tumors is higher than of astrocytic tumors, being associated with the loss of heterozygosity (LOH) on chromosome 1p and 19q [20]. 

### 1.3. Definition of the Clinical Problem

Favorable prognostic factors identified from the randomized radiotherapy trials in LGGs are young age (<40 year), histological subtype of oligodendroglioma, good clinical status at disease onset, seizures as only symptom, and relatively small tumor size [21]. Thus, the total number of unfavorable factors at tumor presentation can be determined and used as a prognostic score for individual patients. Up to two factors identifies low-risk patients, whereas more than two identifies high-risk patients [21]. High-risk patients are identified for immediate postoperative tumor treatment, while low-risk patients are subsided to “watchful waiting”. 

In spite of careful monitoring of symptoms and radiological findings, the assessment of early tumor progression in individual patients may be difficult. LGGs are generally nonenhancing tumors, and standard MRI protocols enhanced with gadolinium show limitations for evaluation of LGGs [22, 23]. Another difficulty is the interpretation of stable disease. It has become clear that from a radiological point of view, there is no such state as stable disease. Volumetric studies by repeated MRI have demonstrated continuous tumor growth during the clinically stable phase of disease [24–26]. Thus, in addition to measuring volumetric tumor growth by MRI, there is a need for imaging techniques that reflect tumor activity. 

Positron emission tomography (PET) can be used to measure the metabolic activity of gliomas and has been useful in various clinical situations. In this paper, the current knowledge on the applications of PET for LGGs is discussed, with focus on amino acid tracers, and recommendations are made for future studies. Literature references were identified through searches of PubMed with the search terms “low-grade glioma”, “PET”, “^11^C-methyl-L-methionine (MET)”, “^18^F-fluoro-ethyl-L-tyrosine (FET)”, ^18^F-fluoro-L-dihydroxyphenylalanine (FDOPA), “radiotherapy”, “chemotherapy”, “prognosis”, “survival”, and “progression” from 1990 until 2010. Articles were identified also through searches of the authors' own files including information from international congress proceedings specialized on clinical neuro-oncology. Only papers published in English were reviewed. 

## 2. Positron Emission Tomography (PET)

Positron emission tomography (PET) has been used for over two decades to image cancer metabolism. The most prominent example is the use of ^18^F-fluorodeoxyglucose (FDG), a radiotracer derived from 2-deoxy-d-glucose, to study the first steps of glucose metabolism. FDG PET is used to stage cancer and to differentiate between malignant and benign lesions [27]. In neuro-oncology, the largest experience has been acquired with FDG PET. Malignant brain tumors are characterized by an increase in glucose consumption and the relationship between histological features and radiotracer uptake is well established [28, 29]. As a consequence, FDG PET is a valuable tool for guidance of stereotactic biopsies in human gliomas [30]. 

The increased protein metabolism in cancer cells compared to normal cells, assessed by radiolabeled amino acids, are other targets for metabolic tumor imaging. ^11^C-methyl-L-methionine (MET), the most frequently studied PET tracer, has a half-life of 20 minutes and is regarded as especially suitable for imaging of brain tumors [31]. The uptake of MET is mainly determined by a specific carrier-mediated mechanism and correlates with the proliferative activity and microvessel density of the tumor cells [32–35]. 

The MET uptake in gliomas is influenced by its specific activity in plasma, the transfer across the blood-brain barrier, the intracellular metabolism, and the incorporation of MET in proteins [36, 37]. Although disruption of the blood-brain barrier is not a prerequisite for increased MET uptake, a damaged blood-brain barrier may enhance leakage of the tracer to the extracellular space and contribute to the increased uptake in malignant gliomas [38]. MET is considered the molecule of choice for gliomas, in spite of its quantification of incorporation which is more difficult compared to FDG [39, 40]. The superiority of MET PET over FDG PET for evaluating glioma is based on the low background uptake of MET in normal brain, providing good contrast with tumor uptake. In accordance, MET is better than FDG in delineating gliomas [40]. However, inter- and intraindividual variations in MET uptake may occur, due to methodological differences but also to competition for the transporter protein by MET and other amino acids [41]. Fasting the patient before PET scan reduces the variability in circulating amino acids, but local variations in MET uptake may still occur, and careful standardization with precise localization of reference regions is important especially for tumors located at the border of gray and white matter [41]. 

The major drawback of MET is its short half-life of only 20 minutes, requiring an on-site cyclotron for production. More recently, the tracer ^18^F-fluoro-ethyl-L-tyrosine (FET) has been established for biopsy guidance and treatment planning of gliomas [42]. The advantage of FET is the longer half-life of 109 minutes, enabling tracer production in a central cyclotron and transport to other units. Although the amino acid is not incorporated into proteins, the influx of FET is mediated by active transendothelial amino acid transport [42]. FET PET measures the magnitude of amino acid transport and its distribution in the tumor. Studies in glioma have suggested similar results for FET PET and MET PET [42–45]. The uptake of MET and FET occurs to a large extent independently of blood-brain barrier disturbance, and shows a very similar uptake intensity and distribution in brain tumors. The experience of FET PET in gliomas is still somewhat limited compared to MET PET.

^18^F-fluoro-L-dihydroxyphenylalanine (FDOPA) is another ^18^F-labeled amino acid analog that has been used for many years to visualize the integrity of the striatal dopaminergic system in patients with movement disorders [46]. FDOPA is brought into tumor cells by amino acid transporters and the tracer can be used also to detect brain tumors [47]. FDOPA was found more accurate than FDG, as well as the nucleoside ^18^F-fluoro-thymidine (FLT) used as a marker for cell proliferation, in detecting LGGs [48, 49]. A correlation between FDOPA uptake and the malignancy grade of newly diagnosed gliomas was recently demonstrated, and future studies may establish a role for FDOPA as a prognostic marker in gliomas [50]. 

## 3. Measurement of Biological Processes by PET

Initial PET studies in gliomas and other cancers were performed as kinetic tracer studies, characterizing time-activity curves of tracer uptake over the entire acquisition period [51]. In the clinical setting, dynamic MET PET studies are not necessary and can be replaced by simpler protocols calculating uptake ratios in the steady state phase of the tracer. With the introduction of the tracer FET, dynamic studies have again received attention, showing increased diagnostic power and prediction of clinical outcome in gliomas [52, 53]. 

**Figure 1:**
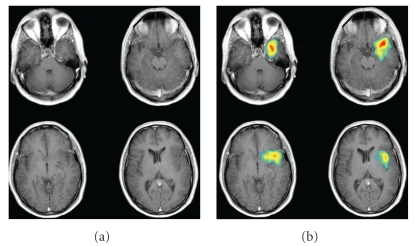
Transaxial postgadolinium T1-weighted MRI (a) and fused MET PET-MRI (b) for spatial correlation of the MET uptake in a glioma in the left frontotemporal region.

Nowadays, PET images are integrated with CT or MR images to map metabolic activity with anatomical regions and structures in the brain (Figure 1). Quantification of tracer uptake by region of interest (ROI) analysis is performed by using a threshold-based algorithm in this area for the lesion itself and for normal brain areas, usually the cortex of the healthy hemisphere or the cerebellum. Tumor volumes are calculated and hot-spot ratios in the tumor, representing areas with highest uptake, by comparing tumor-to-normal brain ratios. In some studies, the parameter “activity tumor volume” is used, defined by calculating tumor volume combined with mean tumor activity [54]. 

Tumor margins of LGGs are often wider estimated by MET than assessed on T1-weighted contrast-enhanced MRI [22, 23, 55]. A direct local comparison of signal changes on presurgical MRI and MET with stereotactic biopsies showed that MET detected solid tumor components as well as infiltration areas with high sensitivity and specificity, providing histological proof for the superior role of MET in delineating tumor extent of gliomas [56]. Interestingly, the infiltrative part of LGGs included in this study showed higher MET uptake compared with the corresponding solid tumor bulk, suggesting that areas of tumor invasion may have a higher demand for amino acids such as methionine [56].

Perfusion studies, using dynamic susceptibility contrast perfusion MRI, in combination with PET have established a positive correlation between MET uptake and tumor vascularity in gliomas [57, 58]. The regional cerebral blood volume (rCBV) of the tumors correlated strongly with MET uptake and was significantly higher in high-grade than in low-grade gliomas [57]. Stereotactic coregistration of CBV and MET showed that both imaging parameters were associated with histopathological features of endothelial proliferation and mitotic activity but not with necrosis [58]. 

Low-grade oligodendrogliomas show a generally higher MET uptake than astrocytomas of similar malignancy grade in spite of their more indolent clinical behavior. Higher MET uptake in oligodendrogliomas is probably correlated to a higher cell density and larger cell turn in oligodendrogliomas compared to astrocytic tumors [59]. Oligodendrogliomas are also known to have a higher microvessel density, consistent with increased CBV values found in oligodendrogliomas [57]. These data demonstrate that high microvessel density in gliomas does not necessarily relate to endothelial cell proliferation. 

## 4. Clinical Applications

**Figure 2:**
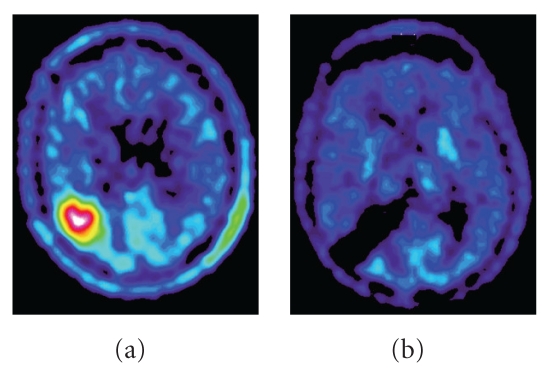
MET PET of a patient with an oligodendroglioma in the right parietal lobe before (a) and after (b) surgical resection and adjuvant radiotherapy, showing no signs of residual tumor at 12 months posttherapy.

In clinical daily practice, functional imaging techniques are not always part of the primary diagnostic setup of patients with suspected LGGs. Instead, they are used when conventional diagnostics fail to give reliable information or are considered too insensitive [51] (Figure 2). In such situations, MET PET may change clinical decisions for patients with brain tumor [68]. In addition to PET, advanced MRI techniques may provide useful information during various stages of disease and improve outcomes for individual patients. Although many of these advanced MR techniques still need to be validated in clinical trials, they are likely to find a complementary role in the management of gliomas in the near future [55]. 

### 4.1. Differentiating Tumor from Nontumor Lesions

MET PET has been used for differential diagnosis of LGGs from other nontumor lesions. Since MET uptake occurs mainly independently of blood-brain barrier disruption, LGGs are generally visualized as hot areas irrespective of malignancy grade. Acute inflammatory reactions in the brain, however, may show increased MET uptake and lead to differential diagnostic problems. The increased MET uptake in acute inflammatory cells is caused by high metabolic rate of these cells and high cell density but probably also by disruption of the blood-brain barrier [69]. 

In spite of the widely accepted view that MET PET can assist in differential diagnosis of tumor from nontumor lesions, only few studies have provided evidence for this clinical application of PET (Table 1). In a consecutive series of 196 patients with suspected brain tumors, differentiation between gliomas and nontumor lesions by MET PET was correct in 79%, using a threshold of 1.47 for MET uptake [60]. Diagnosis was verified by histological examination in 170 patients or by clinical followup and additional investigations (CSF examination or followup MRI) in the remainder [60]. Exclusion of high-grade gliomas reduced the sensitivity to 67%, resulting in 72% specificity for differential diagnosis of LGGs from nontumor lesions. Only 3 out of 31 astrocytomas grade II exhibited lower uptake values than the normal contralateral cortex in this study [60]. In a group of 39 children and young adults (2–21 years) with suspected brain neoplasms, the diagnostic accuracy of MET with regard to differentiating tumors from nontumor brain lesions showed 83% sensitivity and 92% specificity [61]. In a recent study of 88 patients referred to a neurological clinic because of brain lesions, FET PET was shown to detect malignant gliomas with 93% sensitivity and low-grade tumors with 68% sensitivity [62]. For 60 patients, the diagnosis was confirmed by histopathology within one month following PET, for the remaining 28 patients by clinical followup. Two false positive images were found of a total of six postischemic lesions in this study, which was thought related to the slow blood clearance of FET [62]. 

### 4.2. Guiding Stereotactic Procedures and Radiotherapy Planning

As mentioned, there is a correlation in gliomas between MET uptake and tumor histology [51]. Consequently, MET PET has been used for preoperative evaluation of gliomas and, as a further development, for guidance of stereotactic biopsies and radiosurgery [40]. The success rate for PET-guided biopsies was found higher than with CT only [64]. MET PET provided a more sensitive signal compared to FDG PET [30] (Table 1). An increased uptake of both tracers was found in histological samples with anaplasia, but reduced uptake in necrotic areas was shown only by MET [37, 70] (Table 1). Based on these results, MET PET is considered as the molecule of choice for single-tracer PET-guided biopsies in gliomas.

Since MET PET is a valuable instrument for measuring tumor volume, the technique has been successfully used for planning of target volume prior to radiotherapy. It is of specific benefit in LGGs that are ill defined on MRI [63]. This application has been confirmed for FET PET in patients with high-grade gliomas, but no data are available yet for FET and LGGs. In a study of high-grade gliomas, a high interrater agreement was found for biological tumor volumes measured by FET PET-CT [71]. Less consistent measures between observers were demonstrated when using morphological tumor volumes on T1-weighted MRI [71]. It can be concluded that the available evidence supports the role of MET and FET, above other PET tracers, as a complement to volumetric MRI for radiotherapy treatment planning. 

### 4.3. Evaluation of Response to Radiotherapy

MET PET has been evaluated in the followup after radiotherapy but mostly by retrospective reviews including relatively few patients (Table 1). In a postsurgical followup of 30 patients with low-grade astrocytomas, no significant difference in MET and FDG uptake could be detected between tumors with or without adjuvant radiotherapy [65]. Other studies have found a clear decline in mean MET uptake after radiotherapy [66, 67]. These somewhat contradictory results may be explained by different observation times and different radiotherapy modalities used in the study protocols. MET PET was found more suitable than FDG in monitoring therapeutic effects one year after interstitial brachytherapy with ^125^I seeds [66, 67]. Interestingly, the largest decline in MET uptake one year after ^125^I brachytherapy was shown in tumors with high basal MET uptake, suggesting that MET PET can be used as a marker for radiosensitivity in these tumors [67]. 

### 4.4. Evaluation of Response to Chemotherapy

Several reports, all based on limited numbers of patients, have shown a decrease in MET uptake after chemotherapy in LGGs. Reduced MET uptake in hot spots has consistently been reported, but also, reductions of tumor volume were induced by chemotherapy (Table 2). Compared to MRI with fluid-attenuated inversion recovery (FLAIR) technique, MET PET was found more sensitive for the assessment of PCV responsiveness [54]. In a recent prospective study, FET PET was used to evaluate the response to temozolomide in 11 patients with progressive nonenhancing LGGs and compared to MRI [72]. A reduction of FET uptake as early as one month after initiated treatment, preceding MRI volume reductions by several months, was found in some tumors, underscoring the sensitivity of PET with amino acid tracers for detecting early treatment response [72] (Table 2). 

### 4.5. Differentiating Recurrent Tumor from Radionecrosis

Tissue necrosis induced by radiotherapy may cause differential diagnostic problems between treatment effects and recurrent or progressive tumor disease for conventional MRI [55]. Traditionally, FDG PET has been used for this specific application although the low sensitivity of the method has limited its use [80–82]. Chao and coworkers demonstrated that coregistration of FDG PET with MRI increased the sensitivity from 65% to 86% in metastatic brain tumors [83]. More recently, MET PET was shown more successful than FDG PET in differentiating contrast-enhancing areas on MRI induced by radiotherapy from recurrent tumor growth [73, 74] (Table 2). Effective radiation resulted in decreased MET uptake in the tumor, whereas increased MET uptake was an indicator of progressive disease. There was no direct relationship between FDG and MET uptake ratios in histological areas with necrosis, whereas areas with anaplasia showed an increase uptake of both MET and FDG [73]. The generally higher baseline MET uptake in oligodendroglial tumors explained why MET PET provided better diagnostic information with higher sensitivity in astrocytic tumors in this study [73]. 

### 4.6. Long-Term Followup and Prognosis

Volumetric MRI studies have shown continuous growth in LGGs before anaplastic transformation occurs, with predictable growth rate within a relatively narrow range [24–26]. Ideally, PET could be used to measure other parameters in addition to tumor volume that reflect early tumor progression during the time interval prior to accelerated growth on MRI or clinical deterioration [84]. There is indirect support for such a role of MET PET in LGGs, but more robust data are still lacking. One long-term followup study has demonstrated the role of PET in the assessment of time to progression and treatment planning of individual patients [63]. Patients with tumors that showed stable of reduced MET uptake after radiotherapy lived longer in this series [63]. MET PET was shown sensitive in detecting changes in tumor volume over time in LGGs [75]. Combined information on changes in tumor volume measured by MET PET and changes in hot-spot activity over time improved the prediction of time to progression in this retrospective case review [75]. Consistent findings of MET being a sensitive indicator for malignant progression have been reported by Ullrich and coworkers [76]. A significant correlation between changes in MET uptake during tumor progression and the expression of vascular endothelial cells growth factor (VEGF) was found in this study, suggesting that MET uptake may be a surrogate marker for activated VEGF receptor signaling [76]. Baseline uptake of MET at disease onset before treatment has been identified as a prognostic factor for survival in patients with LGGs [77, 78]. 

In a prospective study of LGGs, FET uptake together with tumor growth pattern on MRI was shown to be a strong prognostic factor [79]. Patients with diffusely growing tumors and higher FET uptake ratios had the most unfavorable outcome [79]. A threshold value for FET uptake of 1.1 provided highest prognostic significance, suggesting that the qualitative feature of increased uptake itself is a more important factor for outcome than the absolute value of the uptake ratio [79]. The observation that the tumor growth pattern on MRI, in combination with the FET ratio, has a prognostic impact is an interesting finding and illustrates the importance of imaging tumor behavior in relation to clinical outcome [85]. 

## 5. The Natural History of LGGs

Most PET studies on glioma have focused on measuring MET in tumor areas with highest uptake in relation to clinical parameters, such as tumor histology, response to therapy and patient outcome. Less is known on the spatial changes of MET and FET uptake taking place over time during the natural course of this disease. Monitoring metabolic tumor activity during the evolution of disease, including the number and specific locations of hot spots, is probably a valuable way to study biological tumor behavior. 

**Figure 3:**
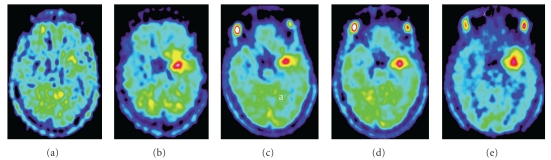
MET PET from disease onset (a) to disease progression (e) of a patient with LGGs in the left hemisphere, showing a gradual increase in hot-spot activity during progressive disease. This patient received radiotherapy between the first (a) and second PET investigation (b), and chemotherapy between the second (b) and third (c) PET investigation. MET PET was performed at approximately 6 months intervals from the time point of disease onset.

**Figure 4:**
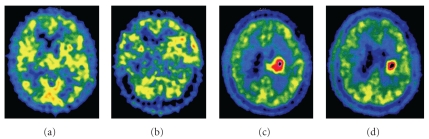
MET PET from disease onset (a) to disease progression (d) of a patient with LGGs in the left hemisphere, showing a shift of weak hot-spot activity located in the left frontotemporal region (a, b), towards a stronger and more centrally located hot spot (c, d) during progressive disease. This patient received radiotherapy between the second (b) and third (c) PET investigation. MET PET was performed at approximately 12 months intervals from the time point of disease onset.

From our own clinical experience of MET PET in patients with LGGs, we have noticed that tumors may show different patterns of progression [75, 86]. In most patients, progressive disease is accompanied by a gradual increase of MET uptake in a preexisting hot-spot, suggesting malignant transformation of this particular tumor area (Figure 3). In other patients, new hot spots arise that may occur prior to clinical and radiological progression and irrespective of tumor treatment. Also, a shift in hot spot from one region to another may be visible during course of disease, suggesting the existence of multiple active metabolic sites within the same tumor (Figure 4). 

One could speculate that tumors comprising several hot spots are biologically more heterogeneous and harbor subclones of tumor cells with different tumor behavior. Since regional molecular heterogeneity is known to be present also in histologically homogeneous glioma types such as LGGs, it is possible that different hot spot regions within one tumor represent different molecular subclones of tumor cells [87]. As a consequence, different tumor areas can show different chemo- and radiosensitivity and MET PET is likely to be a valuable tool to monitor such heterogeneity in response to treatment [88]. 

Support for the existence of spatial heterogeneity in LGGs has come from a PET study, correlating blood flow and FET uptake in a series of LGGs [89]. The majority of tumors included in this study showed an increase of global blood flow in the tumor, measured by ^15^O-H_2_O, together with an increase of FET. Increased blood flow correlated to increased FET uptake and was spatially coupled to the center of the tumor [89]. In individual tumors, however, a spatial heterogeneity was present with regard to the distribution of amino acid uptake and blood flow. Thus, blood flow could be low in spite of high amino acid uptake at the tumor periphery, where infiltration of tumor cells into the peritumoral brain occurs. Low blood flow together with high FET uptake was seen in tumors infiltrating the corpus callosum and could reflect a mismatch between metabolic demands and energy supply, promoting hypoxia [89]. Interestingly, all tumors included in this study appeared as homogeneous nongadolinium enhancing lesions on MRI. 

## 6. Conclusions

LGGs are slowly growing tumors characterized by homogeneous histopathological features but with a large clinical variability in response to treatment and clinical outcome. The management of this patient group requires individual treatment decisions and careful followup of individual patients during the entire course of disease. PET with amino acid tracers, integrated with MRI, is recommended for all patients presenting with a presumed LGGs in clinical centers that have access to PET. This PET examination at the time point of disease presentation is of value for differential diagnosis, to guide stereotactic biopsy, for prognostic assessment, and as a baseline study prior to postoperative therapy. PET may also be used successfully at later clinical time points, when planning for radiotherapy or evaluating response to treatment. The evidence for most of these clinical applications, however, is not strong and mainly based on small retrospective studies. Further developments in this field will largely benefit from larger clinical trials with prospective study designs. In addition, long-term followup of individual patients by PET will provide new insights into tumor behavior in relation to clinical parameters for patients with LGGs. 

## Figures and Tables

**Table 1:** Clinical applications of PET with amino acid tracers in adult low-grade gliomas (LGGs).

Study	Study design	Tumor subtype^1^ no. of patients	Results and/or conclusion
Differential diagnosis tumor versus nontumor lesions			

Herholz et al. [60]	Retrospective review Consecutive suspected tumors Measures: MET ratio, histology	196 patients (99 gliomas grade II)	MET ratio 67% sensitivity and 72% specificity for differential diagnosis grade II gliomas versus nontumor lesions
Galldiks et al. [61]	Consecutive series of children and young adults (2–21 year) with suspected tumors Measures: MET ratio, histology	39 patients (6 AI, 6 AII, 4 AIII, 2 OAIII)	MET ratio 83% sensitivity & 92% specificity for differential diagnosis tumor versus nontumor lesions
Pichler et al. [62]	Retrospective review Consecutive tumors or other intracerebral lesions Measures: FET ratio, MRI, histology	88 patients (19 gliomas grade I and grade II)	FET ratio 94% sensitivity in HGG; 68% sensitivity in LGGs; 2 false positive cases (postischemic lesions) out of 10 nonbiopsy verified inflammatory lesions

Guiding stereotactic procedures and radiotherapy planning			

Goldman et al. [37]	Retrospective review Measures: MET ratio, FDG ratio, stereotactic guided histology	14 gliomas (93 biopsies)	MET ratio correlates with anaplasia; FDG ratio correlates with anaplasia; inverse correlation MET ratio and necrosis; no correlation FDG ratio and necrosis
Levivier et al. [30]	Retrospective review PET-guided radiosurgery Measures: MET volume, MRI	5 LGGs	Spatial accuracy increased by MET volume, especially in ill-defined lesions on MRI
Nuutinen et al. [63]	Prospective long-term followup **** Measures: MET ratio, MET volume, MRI, survival	14 gliomas (13 AII and 1 AIII)	MET ratio and volume: 80% sensitivity in detecting postoperative residual tumor; benefit for radiotherapy planning in 3/14 patients with inconclusive MRI
Pirotte et al. [64]	Retrospective review PET-guided stereotactic biopsy Measures: MET ratio, MET volume, FDG ratio, FDG volume, histology	10 LGGs (6 AII, 2 OII, 1 giant cell astrocytoma, 1 ganglioglioma)	MET ratio corresponded to histology in 9 LGGs; FDG ratio corresponded to histology in 1 LGGs; MET volume superior to FDG volume, especially for cortical tumors

Evaluation of response to radiotherapy			

Roelcke et al. [65]	Postoperative followup of irradiated (*n* = 13) and nonirradiated (*n* = 17) patients Measures: MET ratio, FDG ratio	30 AII	No differences in changes of MET and FDG ratio over time between two groups
Voges et al. [66]	Followup of ^125^I brachytherapy Measures: FDG ratio and volume, MET ratio, and volume	39 gliomas (17 AII, 2 OII, 5 OAII, 1 AI, 2 unspecified, 3 grade III, 8 GB)	Minimal effect of brachytherapy on FGD ratio1 year after seed implantation, but decline of MET ratio
Würker et al. [67]	Follow up of ^125^I brachytherapy Measures: FDG ratio, MET ratio	10 LGGs (2 AI, 5 AII, 2 OAII, 1 OII)	Significant decline in mean MET ratio before and 1 year after brachytherapy; no changes in mean FDG ratio; highest decline rates in tumors with high basal MET ratio

^1^Abbreviations: LGGs: low-grade gliomas; AI: pilocytic astrocytoma; AII: astrocytoma grade II; OII: oligodendroglioma grade II; OAII: oligoastrocytoma grade II; AIII: astrocytoma grade III; OAIII: oligoastrocytoma grade III; GB: glioblastoma.

**Table 2:** Clinical applications of ^11^C-methionine uptake measured by PET in adult low-grade gliomas (LGGs).

Study	Study design	Tumor subtype^1^ no. of patients	Results and/or conclusion
Evaluation of response to chemotherapy			

Tang et al. [54]	Retrospective review Chemosensitivity to PCV Measures: activity volume index (AVI), FLAIR-MRI	7 OII	PCV associated with drastic decrease in AVI; less pronounced decrease in tumor volume on FLAIR-MRI
Wyss et al. [72]	Prospective study TMZ in progressive nonenhancing tumors Measures: FET ratio, FET volume, and MRI	11 LGGs (3 OII, 4 AII, 4 AOII)	Changes in FET preceded and more pronounced than MRI changes; decrease FET ratio < FET volume in responders

Differentiating recurrent tumor from changes induced by radiotherapy			

Van Laere et al. [73]	Retrospective review Differential diagnosis radionecrosis-recurrence Measures: MET ratio, FDG ratio, histology, and survival	30 gliomas (15 LGGs: 8 AAII, 3 OAII, 4 OII)	FDG and MET ratio significant parameters for survival; FDG and MET strongest prognostic accuracy; MET alone strongest prognostic factor for astrocytomas
Terakawa et al. [74]	Retrospective review Differential diagnosis radionecrosis-recurrence Measures: MET ratio (*L/N*_mean_, *L/N*_max_, lesion/normal frontal cortex), histology	77 patients (26 gliomas: 6 grade II, 6 grade III and 14 GB; 51 metastases)	*L/N*_ mean_ with cutoff 1.58 most informative for glioma (75% sensitivity, 75% specificity); *L/N*_max_ not informative for glioma

Long-term followup and prognosis			

Nuutinen et al. [63]	Prospective long-term followup Measures: MET ratio, MET volume, histology, and survival	14 gliomas (13 AII and 1 AIII)	Met ratio and volume: 80% sensitivity in detecting residual postoperative tumor; baseline MET ratio of prognostic value
Ribom et al. [75]	Retrospective review Pretreatment MET Measures: MET ratio, MET volume (untreated versus after surgery/radio- and/or chemotherapy), time-to-progression (TPP)	32 LGGs (11 AII, 6 OAII, 15 OII)	Untreated patients: longest TTP when stable MET ratio and small volume increase; treated patients: initial treatment effects (reduction MET ratio, volume or in both) but no prognostic value
Ullrich et al. [76]	Prospective long-term followup Measures: MET ratio, histology, and molecular tumor profile	24 gliomas (10 AII, 7 OAII, 1 OII, 3 AIII, 3 OAIII)	Mean increase of MET in patients with progression 54.4% versus 3.9% in patients with stable disease; correlation increased MET and VEGF expression
Ribom et al. [77]	Retrospective review Preoperative MET Measures: MET ratio, and survival	89 LGGs (33 AII, 17 OAII, 39 OII)	Preoperative MET ratio prognostic factor for survival in AII and OII
De Witte et al. [78]	Retrospective review Preoperative (*n* = 74) and postoperative (*n* = 11) MET Measures: MET ratio, survival	85 gliomas (28 LGGs: 12 AII, 4 OAII, 12 OII)	MET ratio prognostic factor for survival in grade II and grade III gliomas
Floeth et al. [79]	Prospective followup Histologically verified LGGs Measures: FET ratio, growth pattern on MRI (diffuse versus circumscribed), survival	33 LGGs (27 AII, 2 OAII, 4 OII)	3 major subtypes: (1) low FET ratio and circumscribed on MRI most favorable outcome,(2) positive FET ratio and circumscribed on MRI intermediate outcome,(3) positive FET ratio and diffuse on MRI unfavorable outcome

^1^Abbreviations: LGGs: low-grade gliomas; AI: pilocytic astrocytoma; AII: astrocytoma grade II; OII: oligodendroglioma grade II; OAII: oligoastrocytoma grade II; AIII: astrocytoma grade III; OAIII: oligoastrocytoma grade III; GB: glioblastoma.
